# Fine-scale multiannual survey of benthic invertebrates in a glacier-fed stream used for hydropower generation

**DOI:** 10.1038/s41597-021-00887-x

**Published:** 2021-04-13

**Authors:** Alberto Scotti, Roberta Bottarin

**Affiliations:** Institute for Alpine Environment, Eurac Research, Bozen, Italy

**Keywords:** Environmental impact, Ecosystem ecology, Limnology, Freshwater ecology

## Abstract

The present dataset contains information about aquatic macroinvertebrates and environmental variables collected before and after the implementation of a small “run-of-river” hydropower plant on the Saldur stream, a glacier-fed stream located in the Italian Central-Eastern Alps. Between 2015 and 2019, with two sampling events per year, we collected and identified 34,836 organisms in 6 sampling sites located within a 6 km stretch of the stream. Given the current boom of the hydropower sector worldwide, and the growing contribution of small hydropower plants to energy production, data here included may represent an important – and long advocated – baseline to assess the effects that these kinds of powerplants have on the riverine ecosystem. Moreover, since the Saldur stream is part of the International Long Term Ecological Research network, this dataset also constitutes part of the data gathered within this research programme. All samples are preserved at Eurac Research facilities.

## Abstract (summary)

**Table:** 

Measurement(s)	Abundance of benthic macroinvertebrates • Physical and chemical water parameters • biodiversity assessment objective
Technology Type(s)	Surber sampler • Multiparameter probe • material sampling process
Sample Characteristic - Organism	Aquatic benthic macroinvertebrates
Sample Characteristic - Environment	englacial stream
Sample Characteristic - Location	Province of Bolzano-Bozen • Central Eastern Alps • Italy

Machine-accessible metadata file describing the reported data: 10.6084/m9.figshare.14046554

## Background & Summary

Contributing about 16% of the worldwide electricity production, hydropower is nowadays the largest source of renewable energy^1^. Furthermore, due to the rise in renewable energy request (e.g. for meeting national and international environmental targets), this share is anticipated to increase in the coming decades^2^. Indeed, the International Renewable Energy Agency suggests that current global hydropower capacity (1,308 GW in 2019) would need to increase by 25% by 2030, and by 60% by 2050, to meet the main aim of the Paris Agreement of limiting the global temperature rise to below 2 °C above pre-industrial levels^3^. This ambitious growth rate was accomplished in the five-year term 2015–2019, with an average inter-annual growth in global installed capacity of 2.1%^4^.

A portion of this share is constituted by small hydropower plants (SHPs, definition applying to plants with a capacity of 1–10 MW), whose global installed capacity has increased by approximately 10% from 2013 to 2019^5^. Despite the continuous growth in number of installations, about 66% of the world’s SHPs potential capacity still remains unexploited^5^, thus suggesting that the SHP sector is expected to have a continuous expansion in the near future^6^. Concurrent reasons for this trend are political and economic incentives^7^ and the perception of the general public that “smaller” (compared to large-dam hydropower plants) means a lower social and ecological impact^8^.

However, the notion that SHPs generally constitute a low-footprint technology^9^ is not supported by any strong circumstantial or direct evidence^10,11^, up to the point that some authors argue that the anticipated development of the hydropower sector (including SHPs), may potentially contribute to a deterioration of the ecological status of freshwater ecosystems worldwide^12–14^. Nonetheless, scientific investigation has struggled to follow this course of the hydropower sector, and, despite SHPs amounting to about 91% of currently installed power plants, only 5% of the published studies regarding impact of hydropower energy deals with them^6^. Moreover, in most of these studies, the system design – that can be extremely variable – and the capacity of the SHPs under investigation are not described^15^, making it even more difficult to build thorough scientific knowledge that can contribute to an informed debate on hydropower strategic planning^6^. In addition to that, long-term data for evaluating the effects of SHPs’ activities on riverine ecosystems are chronically lacking. Therefore, current studies are generally based on spatial comparisons with “control” reaches, and the possibility to apply a before-after-control-impact design has so far been precluded^7^.

Here, we provide the dataset *Abundances of benthic invertebrates and related environmental variables over a 5-year sampling period in a glacier-fed stream used for hydropower generation (South Tyrol, Italy)*^16^ collected to evaluate the effect of a small “run-of-river” hydropower plant located on a glacier-fed stream in the Italian Central Eastern Alps. The dataset is composed of 1,991 records and 60 samples (composed by 180 sub-samples) of aquatic macroinvertebrates – widely regarded as very effective bioindicators for riverine environments^17^ – collected in the period 2015–2019, before and after the construction of the SHP, at 6 sampling sites along the stream, twice a year. The samples are available and conserved at Eurac Research facilities, and are part of the faunal dataset collected by the research institution in the frame of the International Long Term Ecological Research (ILTER) programme carried out on the Saldur stream^18^.

## Methods

### Study area

**Fig. 1:**
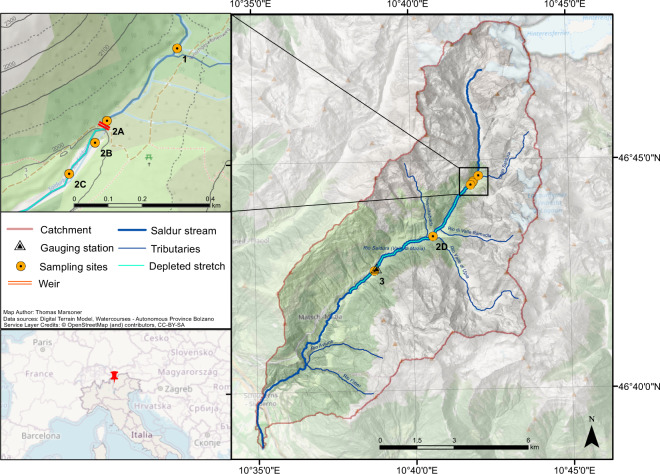
Geographical location of the study area. Map showing the geographical framework of the study area and the location of the sampling sites along the Saldur stream. The upper left map shows a magnification of the geographical area close to the “Tyrolean type” weir of the “run-of-river” hydropower plant.

The Saldur stream catchment – roughly corresponding to the whole Matscher Valley – is located in the Italian Central-Eastern Alps (N 46°, E 10°), in the territory of the Autonomous Province of Bolzano/Bozen, and it drains an area of 101 km^2^ (Fig. 1). The climate in the catchment is relatively dry with an average annual precipitation of about 500 mm, and the Köpper Geiger climate classification for the catchment is spanning from Et to Dfc following a decreasing elevational gradient^19^. The types of land cover in the catchment and their relative and total occupied surface based on CORINE Land Cover data, year 2018 (source: European Environment Agency; http://www.eea.europa.eu) are reported in Table 1. The whole catchment and the Saldur stream have been part of the ILTER network since 2014 (site code IT-25, see: https://deims.org/11696de6-0ab9-4c94-a06b-7ce40f56c964).

**Table 1:** Share and absolute covered surface for each land cover class type throughout the whole catchment (source: CORINE Land Cover data year 2018, European Environment Agency, www.eea.europa.eu).

CORINE Land Cover class types	Share on the catchment	Surface (hectares)
Bare rocks	27.14%	2773.4
Sparsely vegetated areas	24.10%	2463.2
Coniferous forest	21.21%	2167.5
Pastures	6.55%	669.0
Natural grasslands	6.41%	655.3
Transitional woodland-shrub	5.61%	573.3
Glaciers and perpetual snow	4.38%	447.6
Moors and heathland	2.23%	227.9
Non-irrigated arable land	0.88%	89.5
Broad-leaved forest	0.72%	73.1
Land principally occupied by agriculture, with significant areas of natural vegetation	0.34%	34.7
Discontinuous urban fabric	0.29%	29.2
Fruit trees and berry plantations	0.16%	16.2

The Saldur stream, about 21.5 km long, originates from the Matscher glacier and is a tributary of the Adige river, the second longest Italian river. Sectors that rely on Saldur stream’s water are household, tourism, aquaculture but, above all, agriculture and hydropower production^20^.

Indeed, at the beginning of 2016 a small “run-of-river” hydropower plant commenced its operation on the Saldur stream. The SHP is a high-head scheme (456 m from the water diversion to the 5-nozzle Pelton turbine) that implies abstraction without water storage through a “Tyrolean type” weir (located at about 2,000 m a.s.l., weir height: 2 m). The maximum production capacity of the plant is of about 1,600 kW, withdrawing a maximum amount of 900 l/s from the stream. However, 96 l/s are always guaranteed to pass from the upstream to the downstream section of the weir all year round, and from April to October an additional 20% of the measured discharge contributes to the minimum residual flow. A desilting tank that conveys sediments (after desiltation of the water headed to the turbine) about 150 m downstream of the weir is also part of the system. The turbine is located at about 1545 m a.s.l., thus the length of the depleted stretch of the Saldur stream is 7.5 km long. One of the 6 sites (site 1) acts as upstream control, site 2A is located just upstream of the weir, while the remaining 4 sampling points (2B, 2C, 2D, 3) of the dataset are positioned along the depleted stretch.

### Study design

**Fig. 2:**
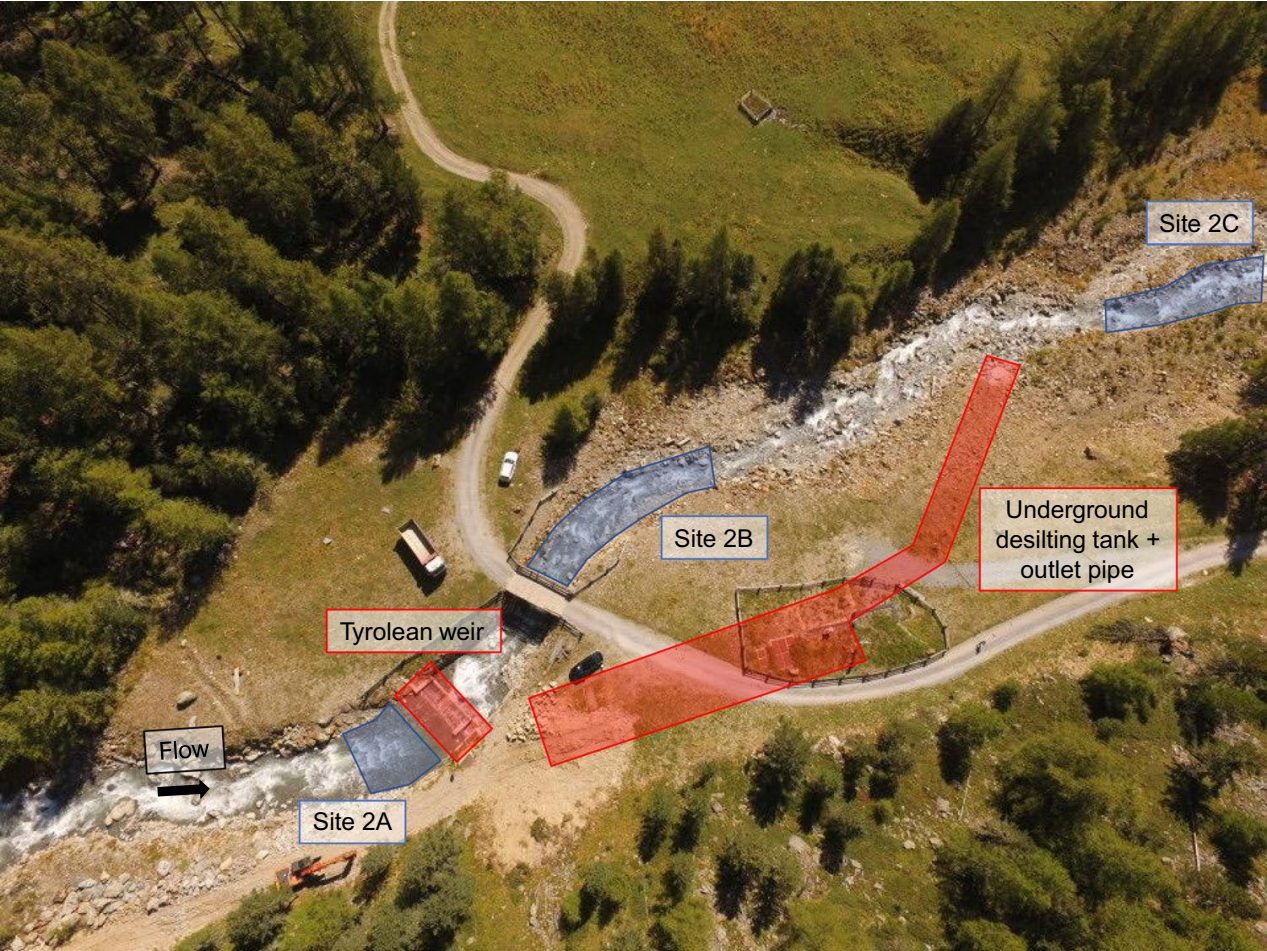
Drone view of the weir area. Photograph showing the relative position of the sampling sites located close to the weir area. Technical features of the “run-of-river” small hydropower plant are represented in red, sampling sites in blue.

The study design was conceived in 2014, when the building operations of the “run-of-river” SHP was planned. Since collection of aquatic macroinvertebrates on the Saldur stream has occurred since 2010^18^, the nomenclature adopted from sampling season 2015 on followed the previous one, resulting in a division of site 2 into four different new sites^21^ while keeping the site nomenclature and position of sites 1 and 3^18,21^. The resulting 6 sites (Fig. 1) – see Table 1 in Scotti *et al*.^21^ for a detailed topographic characterisation of topographic feature of each site – were sampled from 2015 to 2019 twice a year, in late April/mid May, and in late September/mid October. Site 1 acted as the control site, whilst the sites in the weir area, i.e. from 2A to 2C, were selected based on their relative position to the weir and to the outlet pipe of the desilting tank (Fig. 2), and sites 2D and 3 were selected as sites located at about 3 and 6 km downstream of the weir, respectively. Despite monthly sampling campaigns – during glacial melting – performed for the ILTER activities still having been in place^18^, the choice of time of sampling for the present dataset is motivated by the fact that in April/May and September/October the glacial contribution to the total water flow is lower compared to the other spring and summer months^22^, thus resulting in a proportionally higher amount of water diverted by the SHP (calculated on the total water discharge). Higher amounts of diverted water potentially mean a greater impact on the stream biota^23^.

### Fieldwork and laboratory work

We performed a quantitative sampling using a Surber sampler (22 × 23 cm, mesh-size 500 µm). We covered about 20–50 m segment of the stream at each site, and 3 different cumulative samples made up of 4 individual Surbers were collected and pooled, constituting one sub-sample each. The three sub-samples – always kept separated during the sorting, identification, and data processing phases – made up one sample, so that a total of 12 Surber samples were collected at each site, for each sampling event. This choice was motivated by the highly uniform substrate of the stream, mainly composed of macrolithal and mesolithal, and by the fact that a clear division of habitats – e.g. between riffles and pools – did not exist, due to the high-gradient and torrential waters of the stream. Once the substrates to be targeted for each sampling had been identified, the sub-samples were distributed proportionally to the area covered by each substrate. The sampling effort consisted in perturbating the substrate in front of the Surber sampler for about 1 minute. Thus, the final sampled area for every sub-sample was approximately 0.20 m^2^, and 0.60 m^2^ for the entire sample. Each sub-sample was then filtered in the field using a 100 µm sieve-net, preserved in plastic sealed bottles containing 70% ethanol, labelled and brought to the laboratory within the same day.

Concurrently, suspended solids (ml/l) after 30 minutes of water sedimentation in a Imhoff cone, specific conductance at 25 °C (mS/cm, with probe Cond 7, XS Instruments, calibrated with a standard solution of 84 µS/cm), water temperature (°C, with an alcohol thermometer), and the calculation of the bottom component of the Pfankuch channel stability index^24^ were measured and performed. In 2018 only, turbidity (NFU) was measured instead of suspended solids with a dedicated probe (HI9829, Hanna Instruments).

In the laboratory, each sub-sample was sorted without organising sub-fractions, and organisms were separated from the debris. All the organisms present in the sub-samples were then counted and identified to the lowest possible taxonomic level under a stereoscopic microscope at 50 × magnification, referring to appropriate literature^25–33^.

### Data processing

Organisms of each sub-sample were stored in PE bottles containing 70% ethanol, labelled and preserved at Eurac Research facilities, and are available upon request.

The resulting taxon list was digitised and inserted in an internal faunal database of Eurac Research that also contains specific details about locations and concurrent data of environmental variables. After standardising the sampled surface to 1 m^2^ for each sub-sample, the resulting dataset was published through PANGAEA® Data Publisher (https://www.pangaea.de).

## Data Records

The dataset *Abundances of benthic invertebrates and related environmental variables over a 5-year sampling period in a glacier-fed stream used for hydropower generation (South Tyrol, Italy)*^16^ is hosted by PANGAEA® Data Publisher and can be download as tab-delimited text using different character encodings.

**Fig. 3:**
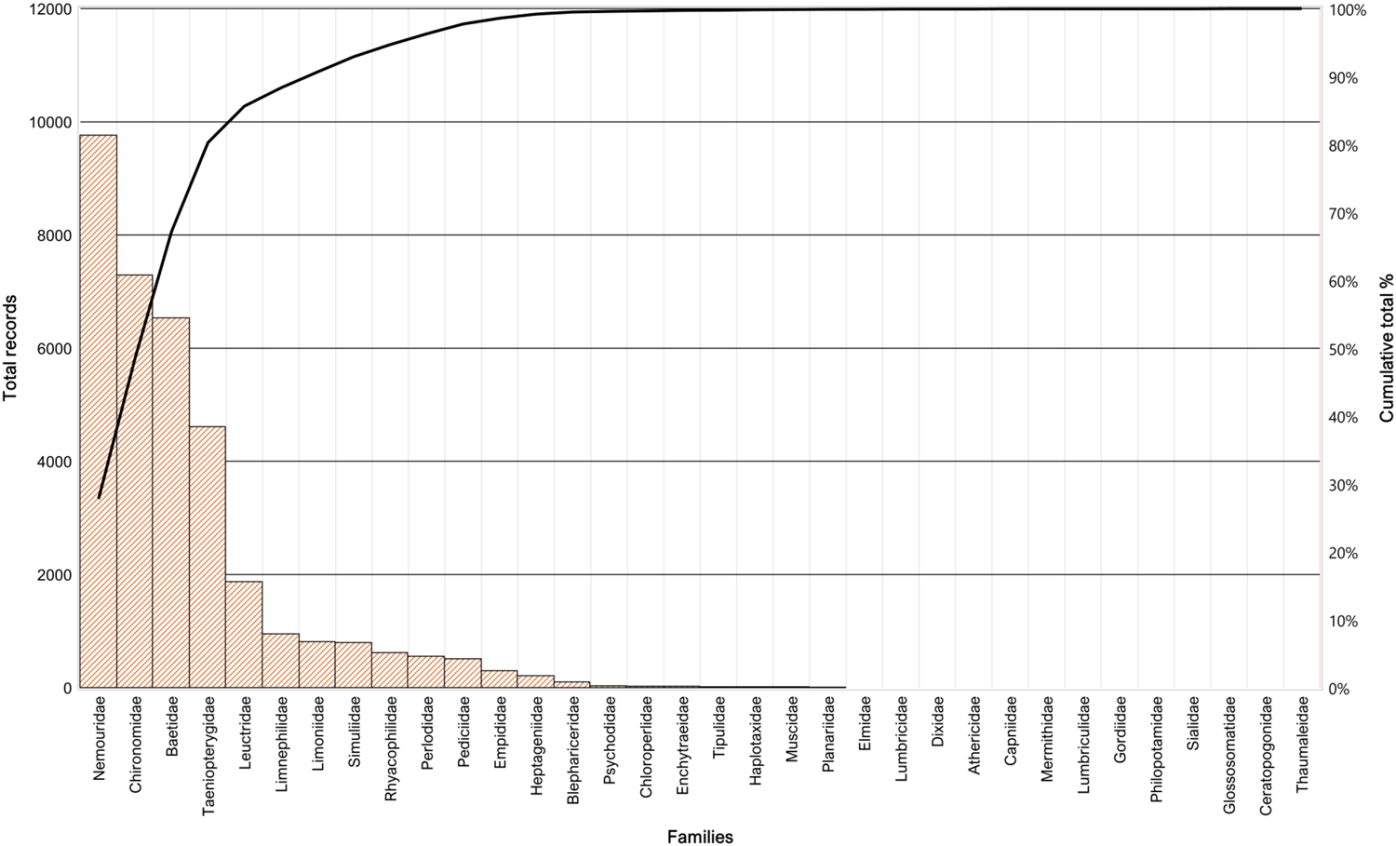
Number of occurrences per family. Pareto chart showing organisms of the dataset grouped by family, and their relative contribution to the total number of collected organisms.

The dataset is composed by 1,991 records for the faunal part, and 156 records for the environmental variables part. The faunal part of the dataset contains data coming from 60 samples and 180 sub-samples. 34,836 organisms were collected (Fig. 3), 30% of which identified at species level, 48% at genus level, and 22% at family or subfamily level. About 90% of the total number of organisms retrieved fell within 6 macroinvertebrate families (Nemouridae, Chironomidae, Baetidae, Taeniopterygidae, Leuctridae, Limnephilidae), representing species typically associated with glacier-fed streams (Fig. 3). All records of the dataset are georeferenced and defined by a date. For the environmental variables, also hour of sampling is provided.

## Technical Validation

All the sampling events were performed with the presence of at least one of the authors. The taxonomic identification was carried out by the same author (A.S.), who also supervised and carried out the sorting process.

The taxonomic nomenclature fully complies with “Fauna Europaea” standards^34^, and consistency of the dataset in terms of time, space, and possible taxonomic incongruences was checked and reviewed by Dr. Daniela Ransby, data curator for PANGAEA® Data Publisher.

## Data Availability

No custom code has been used during the generation and processing of this dataset.
